# Effects of a Session of Exergames and Traditional Games on Inhibitory Control in Children With Autism Spectrum Disorder: Randomized Controlled Crossover Trial

**DOI:** 10.2196/65562

**Published:** 2025-03-05

**Authors:** Juliana Macedo Miranda, Rodrigo Alberto Vieira Browne, Weslley Quirino Alves da Silva, João Paulo Rodrigues dos Santos, Carmen Silvia Grubert Campbell, Isabela Almeida Ramos

**Affiliations:** 1 Graduate Program in Physical Education, Catholic University of Brasilia, QS 07 Lote 01 EPCT Águas Claras, Brasilia, 71966700, Brazil, 55 61991101717

**Keywords:** children, pediatric, autism, ASD, autistic, behavior, exergame, physical education, exercise, physical activity, cognition, anthropometric, Flanker test, inhibitory control, randomized control trial, crossover

## Abstract

**Background:**

Autism spectrum disorder (ASD) is characterized by deficits in executive functions, such as inhibitory control, which affect behavior and social adaptation. Although physical activity–based interventions, such as exergames, have shown potential to improve these functions, their comparative effects with active traditional games remain underexplored, particularly regarding inhibitory control in children with ASD.

**Objective:**

We aim to analyze the effects of a session of exergames and active traditional games on inhibitory control in children with ASD.

**Methods:**

This randomized controlled crossover trial included 9 male children with ASD (mean age 8.6, SD 1.4 y). Participants completed three 20-minute experimental sessions in random order, with a minimum interval of 48 hours: (1) active traditional games, (2) exergames using Just Dance 2022, and (3) a control session with manual painting activities. Inhibitory control was assessed 5 minutes postsession using a modified flanker task in the E-Prime (version 3.0; Psychological Software Tools Inc) program, recording reaction time (RT) and accuracy in congruent and incongruent phases. Repeated measures ANOVA was used to compare RT and accuracy between experimental and control conditions. Data are presented as means and 95% CIs.

**Results:**

There was a statistically significant effect of condition on RT in the incongruent phase (*P*=.02). RT in the exergame session (849 ms, 95% CI 642 to 1057) was lower compared to the traditional games (938 ms, 95% CI 684 to 1191; *P*=.02) and control (969 ms, 95% CI 742, 1196 to *P*=.01) sessions. No significant differences were observed in RT during the congruent phase or in accuracy across either phase.

**Conclusions:**

A 20-minute session of exergame improved inhibitory control performance in children with ASD compared to active traditional games and painting activities.

## Introduction

Autism spectrum disorder (ASD) is characterized by significant challenges in reciprocal social communication and interactions, as well as restricted and repetitive behavioral patterns [1,2]. These challenges are closely associated with pronounced impairments in executive functions, particularly inhibitory control, which is linked to deficits in social communication [3] and repetitive behaviors [4]. Moreover, impairments in inhibitory control and working memory in children with ASD are associated with low school readiness [5]. Inhibitory control plays a crucial role in academic performance by regulating attention, behavior, and thought [6] and managing emotions to overcome internal impulses or external stimuli, thereby enabling adaptation to challenging contexts [7]. This highlights the importance of investigating inhibitory processes in ASD and the factors that may influence their outcomes.

Studies have demonstrated the positive effects of physical exercise on inhibitory control in both neurotypical children [8] and children with ASD [9]. For instance, a study involving children with ASD conducted three 20-minute exercise sessions—circuit training, treadmill walking, and a sedentary control—and observed significant improvements in cerebral oxygenation and inhibitory control during the exercise sessions [9]. These acute benefits of exercise are attributed to the release of catecholamines and neurotrophic factors [10], as well as enhanced cerebral oxygenation regulation, which improves cognitive processing [9,11]. However, many children fail to meet recommended levels of physical activity [12,13], and children with ASD are particularly less likely to engage in school-based movement activities than their neurotypical peers [14]. In light of the World Health Organization’s campaign emphasizing that “every move counts,” as long as it is enjoyable, accessible, safe, and valued [15,16], exploring alternative strategies to promote physical activity and assess their potential effects on inhibitory control in children with ASD is essential.

Playful activities, such as traditional games, can effectively encourage physical activity and enhance inhibitory control during classroom time [17]. However, it remains unclear whether exercise sessions based on play produce similar effects on inhibitory control in children with ASD. In Brazil, physical education classes, which are integral to the school curriculum, play a crucial role in child development by fostering exploration of emotions, reasoning, imagination, and creativity through play and games [18,19]. Yet, children with ASD often struggle to participate in symbolic play due to delays in behavioral skills such as communication and socialization, as well as learning barriers such as rigidity and repetitive behaviors [20]. These challenges may result in social isolation and limit opportunities to benefit from traditional Physical Education alongside peers [21,22]. This underscores the need to adapt school physical education sessions to accommodate the behavioral needs of children with ASD and explore alternative practices that provide comparable benefits for executive functioning, particularly inhibitory control.

Exergaming, which integrates physical exercise with digital games, has emerged as a promising strategy to engage individuals in activities combining physical and cognitive stimulation, aligning with educational and therapeutic goals [23,24]. These interventions address the unique challenges faced by children with ASD, offering an enjoyable and accessible medium to foster motor and executive skills [25]. Platforms such as Dance Dance Revolution have garnered attention for merging physical activity with cognitive engagement in an interactive format. Studies indicate that such platforms enhance cognitive performance, including executive functions, while also providing physiological and motivational benefits [26]. Features such as dynamic adaptation to movement and immediate feedback make exergames particularly relevant in educational and therapeutic contexts. This study specifically used Just Dance 2022 due to its ability to adapt dynamically to users’ movements, providing immediate visual and auditory feedback, which can enhance engagement and motivation. The game’s structure, which includes a wide range of songs with varying levels of difficulty, allows participants to select options that align with their abilities and preferences, promoting inclusion and reducing potential barriers to participation. These features make Just Dance 2022 an ideal platform for addressing the unique behavioral and cognitive challenges faced by children with ASD.

To address the need for further robust evidence on exergame interventions in school settings, this study aimed to analyze the acute effects of a session of exergames, specifically Just Dance 2022, and active traditional games on inhibitory control performance of school-aged children with ASD. By exploring these 2 distinct approaches, this study seeks to provide contributions to alternative strategies for promoting executive functioning and physical activity among children with ASD, particularly in environments such as schools, where inclusive and engaging practices are essential.

## Methods

### Study Design

This randomized controlled crossover trial was registered in the ReBEC (Brazilian Registry of Clinical Trials; protocol RBR-5r9xzbq). This study was conducted in accordance with the CONSORT (Consolidated Standards of Reporting Trials) guidelines for randomized clinical trials [27]. Given the within-subjects crossover design, measures were implemented to address potential day-to-day variations and carryover effects in performance. The 48-hour washout period between sessions was selected based on the available literature, including studies involving children with ASD and typically developing children, which demonstrated the adequacy of this interval to minimize residual effects [8,9]. Additionally, randomization of the session order was employed to counterbalance potential biases introduced by session sequencing. All sessions were conducted at the same time of day for each participant, in a controlled environment, and by the same research team to ensure consistency.

### Ethical Considerations

Ethical approval was obtained from the Ethics Committee on Human Research at the Catholic University of Brasília (protocol 5448930/2022). Prior to participation, informed consent was obtained from parents or legal guardians, who received a detailed explanation of the study’s objectives, procedures, potential risks, and benefits. In addition, assent was obtained from the child participants, ensuring their voluntary participation. To ensure participant privacy and confidentiality, all collected data were fully anonymized and stored securely. No financial or material compensation was provided, as the study adhered to Brazilian ethical guidelines, which prohibit compensation for research participation. The study was conducted in accordance with the Helsinki Declaration and Resolution 466/2012 of the Brazilian National Health Council.

### Randomization

Participants were randomly assigned to the experimental and control sessions to minimize bias and enhance the reliability of the results. Randomization was conducted by a blinded researcher who was not involved in data collection, ensuring impartiality. The allocation list was generated electronically using the GraphPad website [28], guaranteeing a rigorous random selection process. The exercise protocol was pretested in a pilot study and was subsequently implemented with a 48-hour interval between sessions. The protocol included (1) an exergame session, (2) a physical education session with active traditional games, and (3) a control session with manual painting activities.

### Participants

Nine male Brazilian schoolchildren were recruited from Escola Classe 10, a public elementary school located in the administrative region of Taguatinga, Federal District, Brazil, to participate in this study. The inclusion criteria were: children aged 6 to 11 years, enrolled in school, diagnosed with ASD according to the *DSM-5* (*Diagnostic and Statistical Manual of Mental Disorders, Fifth Edition*) [29], and without a history of physical, pulmonary, or cardiac malformations. The exclusion criteria included nonverbal ASD, intellectual disability (IQ below 70), and voluntary withdrawal from this study. Additionally, parents or guardians provided the child’s medical history.

### Procedures

The evaluations and interventions were conducted at the premises of Escola Classe 10. Data collection took place between August and October of 2022. The first meeting involved anthropometric measurements and the administration of the Körperkoordinationstest für Kinder (KTK) battery [30]. In the second meeting, Raven Colored Progressive Matrices Test was applied [31,32]. The third meeting was dedicated to familiarizing participants with the flanker task. The final 3 meetings involved the exergame, traditional games, and control sessions, each followed by the application of the flanker task. A cognitive test was administered within 5 minutes after each session. The sessions were carried out during the school hours of each child, in the respective shift (morning or afternoon) in which they were enrolled.

### Initial Assessments

#### Anthropometry

Weight and height were measured using an electronic scale (Tec-Silver, Techline) and a fixed wall stadiometer (Sanny ES2040, Sanny), respectively, following the procedures outlined by Heyward et al [33]. The BMI was calculated by dividing weight (in kilograms) by the square of height (in meters, kg/m²). The calculations were performed according to the guidelines provided by the Centers for Disease Control and Prevention [34].

#### Body Coordination Test

The motor skills of the participating children were assessed using the body coordination test for children (KTK). The KTK is a standardized instrument designed to evaluate various aspects of body coordination. The test battery includes 4 tasks—balancing, jump on one foot, lateral jump, and lateral transposition—each targeting specific components of body coordination, such as balance, rhythm, strength, laterality, speed, and agility. These tasks collectively aim to assess the overall body coordination and mastery of movement [30]. The final score of the KTK is obtained by summing the points from the 4 tasks, which are compared to normative data for the age group. Based on performance, participants were categorized into 2 groups: normal coordination for those within the expected range of motor skills, and nonnormal coordination for those whose results indicated potential delays or difficulties in motor development.

#### Raven Colored Progressive Matrices Test

The Raven Colored Progressive Matrices test, a nonverbal intelligence assessment, was employed to evaluate cognitive abilities. The test consists of 36 items divided into 3 series (A, Ab, and B), each containing 12 items. The total score is the sum of the correct answers across all 3 series, with series B being more difficult than Ab, and Ab being more difficult than A. Each item presents a figure with a missing part, and the child must select the correct option from 6 alternatives to complete the figure. One point was awarded for each correct answer, and 0 for incorrect answers, yielding a total score range from 0 to 36 points. The first section of the test involves completing missing parts of continuous patterns, focusing on identical or sequential patterns. Series Ab and B involve tasks related to analogies, permutations, pattern alterations, and logical relationships, with no time limit for completion [31,32]. The raw score was calculated by summing the correct answers. This score was then converted into a percentile based on the child’s age and total points. The conversion of the raw score into percentile and standard scores enabled the interpretation of the child’s intelligence level.

### Experimental and Control Sessions

Three types of sessions were conducted: the exergame session with Just Dance 2022, the active traditional games session, and the sedentary control session. The first 2 sessions involved physical activity, while the control session was sedentary. All sessions were conducted with the participants’ classmates, totaling 17 students per class, which included 15 typically developing children and 2 children with ASD. However, only the children with ASD were evaluated. Each session was administered by 2 researchers with assistance from the classroom teacher and the monitor assigned to children with ASD, totaling 4 facilitators.

The Just Dance 2022 exergame session was conducted in an air-conditioned classroom at the school. The Xbox One console, Kinect sensor, and Samsung TV (model UN32J4290) were used for the intervention. The Kinect sensor and TV were positioned 0.8 m from the ground, and the player stood at least 1.8 m from the television. The session involved 5 songs, totaling 20 minutes and 50 seconds of activity, with 15-second breaks between each song, bringing the total session time to 22 minutes and 5 seconds. Songs were selected based on their difficulty level (from easy to difficult; for more details, see Table S1 in Multimedia Appendix 1). Just Dance 2022 is a digital dance game that recognizes body movements through Kinect (Xbox) and presents various dance movements with different levels of difficulty, resembling moderate to high-intensity interval exercise. Players are scored based on the accuracy of their movements; the closer the player’s movements align with the ones shown on the screen, the higher the score.

The active traditional games session was held on the school’s multisport court and included 4 games: statue of the body parts, save yourself with a hug, hula hoop dance, and even-odd tag (for more details, see Table S2 in Multimedia Appendix 1). These games required various perceptual-motor skills, such as coordination, balance, and laterality, and emphasize cooperation and respect for diversity. The activities were taught according to the Movement Curriculum of the Federal District for Elementary School Initial and Final Years [35]. The session lasted 20 minutes, and a JBL Flip 4 Bluetooth speaker, along with 15 hula hoops, was used to support the games.

The sedentary control session was applied in an air-conditioned classroom. In this session, the children engaged in a quiet activity of coloring superhero pictures for 20 minutes. No physical activity was involved, and no heart rate measurements were taken during this session.

### Heart Rate Monitoring

Heart rate was measured during the experimental sessions using a Galaxy Watch4 BT 40mm smartwatch. Due to the reluctance of several children with ASD to wear a chest strap, the original plan to use a Polar FT1 heart rate monitor was adjusted. Instead, heart rate was recorded at 5-minute intervals during the sessions (at 5, 10, 15, and 20 min of exercise). The mean heart rate was calculated from all recorded readings, and the percentage of maximum heart rate (HRmax) was assessed during the sessions. Activity intensity was determined based on HRmax, calculated using the Tanaka formula: 208 - 0.7 × age [36]. Although the Galaxy Watch4 smartwatch has not been specifically validated for children with ASD, studies have demonstrated its validity for monitoring heart rate during physical activity in young adult populations [35,37].

### Inhibitory Control Task

**Figure 1.**
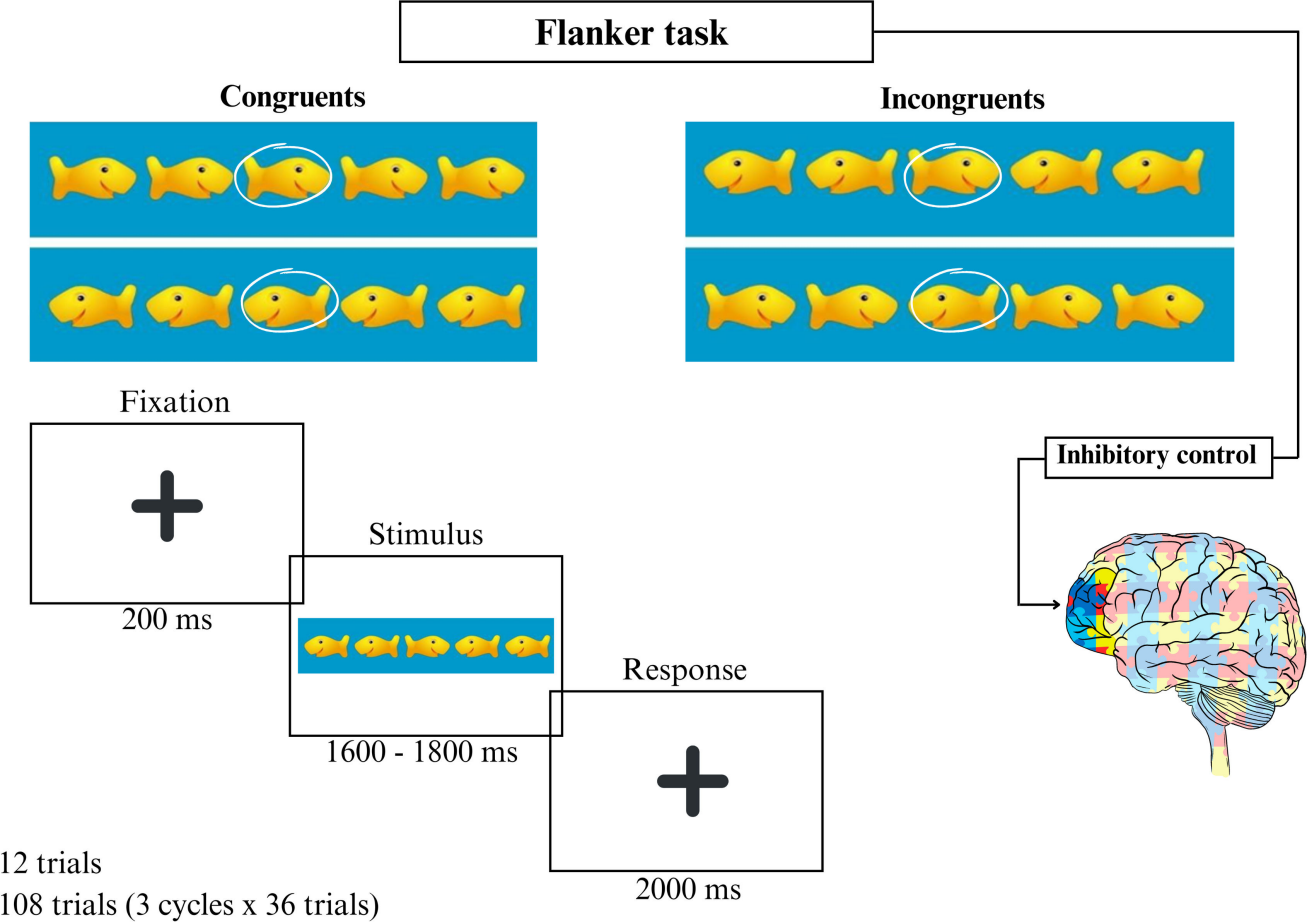
Illustration of the modified flanker task. Top panel: Possible combinations of congruent conditions (fish facing the same direction) or incongruent conditions (central fish facing the opposite direction). Bottom panel: Sequence of events and duration of each stimulus.

A modified version of the Eriksen flanker task [38,39] was used to assess attentional inhibition. In this task, the stimuli consisted of visual representations of fish, with a target fish presented at the center, facing either to the right or left. The child’s attention was directed toward the target fish, surrounded by 4 flanker fish on either side. The flanker fish, which needed to be ignored, could either face the same direction as the target (congruent condition) or the opposite direction (incongruent condition). Children were instructed to indicate the direction of the target fish by pressing the corresponding keys (← for left-facing target fish and → for right-facing target fish). The task consisted of 2 types of trials: congruent, where the direction of the flanking fish matched the target fish, and incongruent, where the direction of the flanking fish was opposite to the target fish. The experiment took place in a room with ambient light and air conditioning, and children were positioned in front of a computer programmed with the E-Prime (version 3.0; Psychological Software Tools Inc) software to present the stimuli. Before the test, the examiner explained the procedure, and a familiarization session was conducted with 12 trials (3 trials of each type: congruent and incongruent for both right- and left-facing target fish). Following the familiarization session, the experimental task was administered, consisting of 108 test trials presented in 3 blocks (36 trials per block). The trials were randomly assigned to include an equal number of congruent and incongruent trials, with the target fish presented randomly on the left or right. Each test began with the presentation of a central fixation point, followed by the display of 5 fish (2.5 cm in size) on a blue background for 200 ms. The stimuli remained on the screen until a response was made or for up to 2000 ms. The intervals between stimuli were randomly varied at 1600, 1700, or 1800 ms. The design followed previous studies using the flanker task to analyze inhibitory control in children with ASD [40-42]. For each trial, children were instructed to respond as quickly and accurately as possible by pressing a key to indicate the direction of the central target fish (left or right; Figure 1). The analyzed data included reaction time (RT) in milliseconds and the percentage of correct responses (accuracy) for both congruent and incongruent phases.

### Data Analysis

The descriptive statistics for sample characteristics were presented as mean (SD; for continuous variables) and as absolute and relative frequencies (for categorical variables). The normality of the data distribution was assessed using the Shapiro-Wilk test. Repeated measures ANOVA was conducted to compare RT and accuracy between the experimental and control sessions. Following a significant ANOVA, post hoc analyses using the least significant difference test were performed. The assumption of homogeneity of variance was assessed with Levene test. To improve the reliability of the results and correct for deviations from normality, bootstrapping procedures with 1000 resamples were applied, and 95% CIs for the differences between means were obtained. Descriptive ANOVA results were reported as means and 95% bootstrapping CIs. Effect size was calculated using partial eta squared (η^2^), and Cohen *d* was computed for pairwise comparisons in the post hoc analysis. Paired 2-tailed *t* tests, also conducted with bootstrapping procedures with 1000 resamples, were used to compare heart rate between experimental sessions. A *P*<.05 was considered statistically significant. All statistical analyses were performed using SPSS for Windows (version 27; IBM Corp).

## Results

### Sample Characterization

The sample characterization is summarized in Table 1. The mean age was 8.6 (SD 1.4) years, and the BMI indicated a wide range of nutritional statuses, including thinness (n=2, 22.2%), normal weight (n=3, 33.3%), and overweight or obesity (n=4, 44.4%). Motor coordination, assessed by the KTK, was below the normative range in 33.3% (n=3) of the participants. Cognitive assessment using the Raven test revealed a mean score within the expected range for this age group. Comorbidities were prevalent, with 44.4% (n=4) of the children diagnosed with attention-deficit/hyperactivity disorder, 11.1% (n=1) presenting with microcephaly, and 11.1% (n=1) with central auditory processing disorder. Psychological support was the most common (n=7, 77.8%), followed by speech therapy (n=5, 55.6%) and occupational therapy (n=3, 33.3%). Other forms of support included education assistance (n=3, 33.3%), psychomotricity (n=2, 22.2%), and, less frequently, music therapy (n=1, 11.1%) and ecotherapy (n=1, 11.1%).

**Table 1.** Characterization of children with autism spectrum disorder (N=9). The data are presented as mean (SD; continuous variables) and absolute and relative frequencies (categorical variables).

	Values
Age (years), mean (SD)	8.6 (1.4)
Weight (kg), mean (SD)	35.7 (14.4)
Height (cm), mean (SD)	1.38 (0.12)
BMI (kg/m^2^), mean (SD)	18 (4.9)
Thinness, n (%)	2 (22.2)
Normal weight, n (%)	3 (33.3)
Overweight or obese, n (%)	4 (44.4)
KTK^a^ score, mean (SD)	95.8 (16.5)
Normal coordination, n (%)	6 (66.7)
Nonnormal coordination, n (%)	3 (33.3)
Raven^b^ score, mean (SD)	111.7 (16.6)
Comorbidities, n (%)	
Microcephaly	1 (11.1)
ADHD^c^	4 (44.4)
CAPD^d^	1 (11.1)
Therapeutic approaches, n (%)	
Psychologist	7 (77.8)
Speech therapist	5 (55.6)
Occupational therapist	3 (33.3)
Education support	3 (33.3)
Psychomotricity	2 (22.2)
Nutritionist	1 (11.1)
Music therapy	1 (11.1)
Ecotherapy	1 (11.1)

a KTK: Kӧrperkoordinationstest für Kinder (body coordination test for children).

b Raven Colored Progressive Matrices test.

c ADHD: attention-deficit/hyperactivity disorder.

d CAPD: central auditory processing disorder.

**Figure 2.**
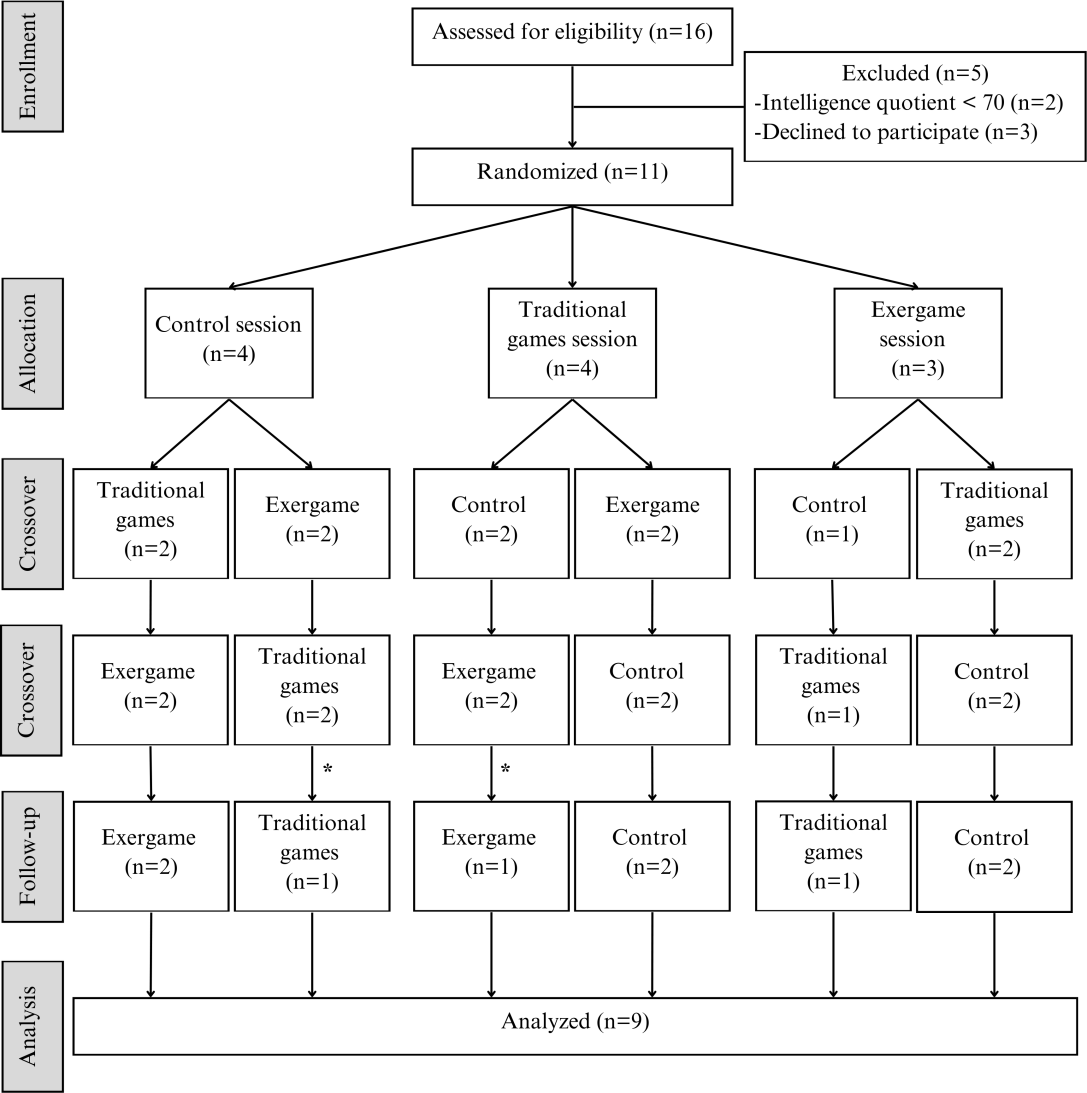
Study flowchart. *Loss of follow-up.

Figure 2 depicts this study’s flowchart, including all 3 sessions—exergame, traditional games, and control—and their respective order of implementation. Each participant completed all 3 conditions in a randomized sequence, with a 48-hour washout period between sessions to mitigate carryover effects and ensure data reliability.

### Heart Rate Monitoring

Heart rate during the active traditional games session (153.8 bpm; 95% CI 139.4 to 168) showed a trend toward higher values compared to the exergame session (129 bpm, 95% CI 121.8 to 135.6), with *P*=.052. Similarly, the active traditional games session reached higher values of HRmax (76.1%, 95% CI 69.1 to 83.3) compared to the exergame session (63.9%, 95% CI 60.3 to 67.2), with *P*=.053. Both conditions reached moderate intensity.

### Inhibitory Control Task

**Figure 3.**
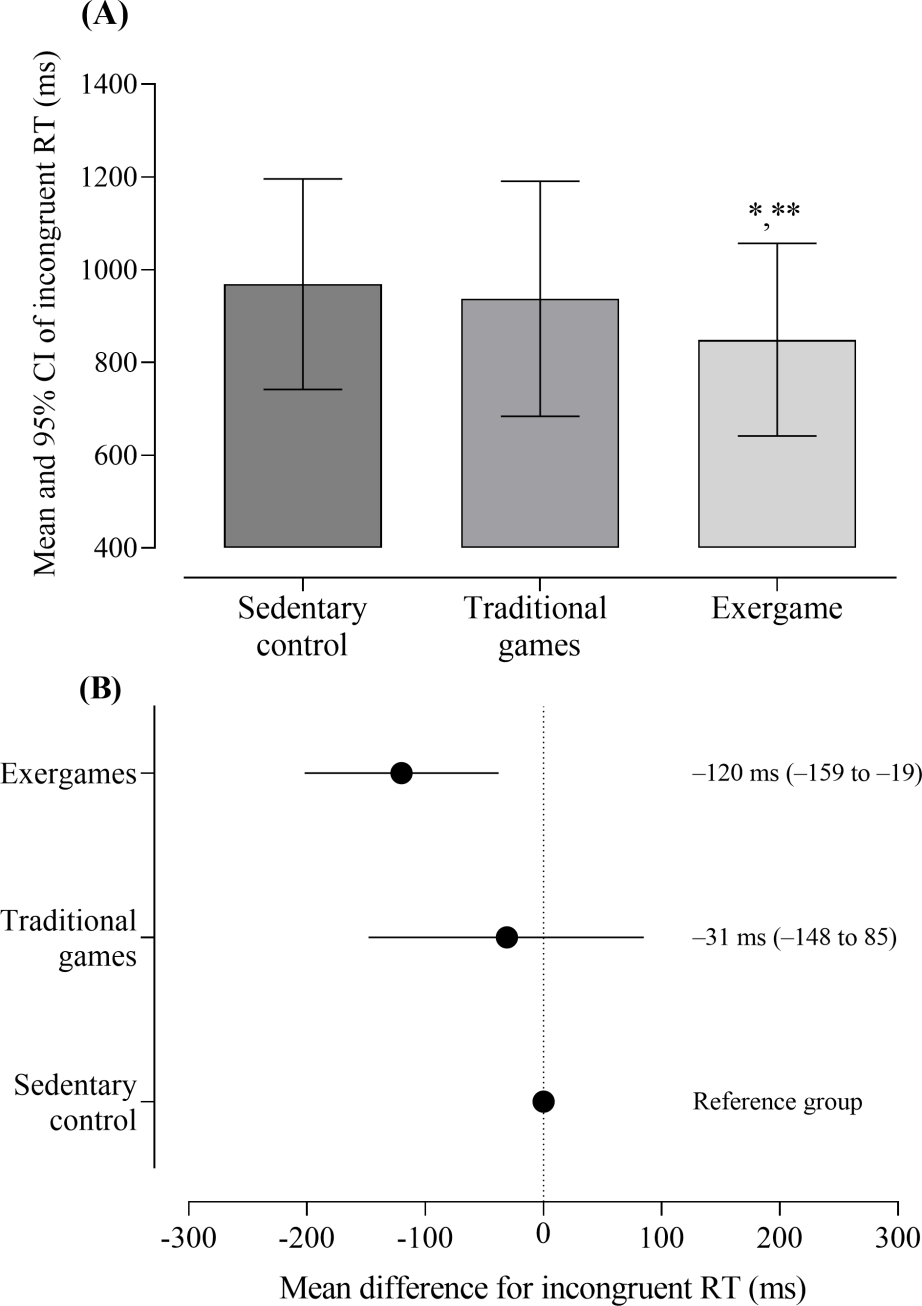
Effects of a session of exergames and traditional games on inhibitory control performance, specifically on incongruent RT, in children with autism spectrum disorder (N=9). (A) Mean values and CIs for incongruent RT in each experimental and control session. (B) Mean difference of the exergames and traditional games sessions compared to the reference session (sedentary control). The data are presented as mean, mean difference, and 95% CI. *P=.01 compared to the control session. **P=.02 compared to the traditional games session. RT: reaction time.

Table 2 and Figure 3 presents the results of RT and accuracy for the congruent and incongruent phases between the experimental and control sessions. A statistically significant effect of the condition was found for incongruent RT (*P*=.02; η^2^=0.38). Post hoc analysis revealed that the exergame session resulted in a shorter RT compared to the control session (849, SD 270 vs 969, SD 295 ms; mean difference: −120 ms, 95% CI −202 to −38; *P*=.01; Cohen *d*=−1.1, 95% CI −2.0 to −0.3), as well as compared to the traditional games session (849, SD 270 vs 938, SD 330 ms; mean difference: −89 ms, 95% CI −159 to −19; *P*=.02; Cohen *d*=−1.0, 95% CI −1.8 to −0.2). No statistically significant effects were found for congruent RT, nor for congruent and incongruent accuracy (*P*>.05).

**Table 2.** Effects of a session of exergames and traditional games on inhibitory control performance in children with autism spectrum disorder (N=9). The data are presented as mean, 95% bootstrapping CI, and partial eta squared (η^2^).

	Control session, mean (95% CI)	Traditional games session, mean (95% CI)	Exergame session, mean (95% CI)	*P* value	η^2^
Congruent					
Reaction time (ms)	879 (673 to 1085)	829 (623 to 1035)	775 (571 to 978)	.08	0.27
Accuracy (%)	90.9 (77.7 to 104.2)	87.9 (73.1 to 102.6)	89.1 (77.2 to 101)	.30	0.14
Incongruent					
Reaction time (ms)	969 (742 to 1196)	938 (684 to 1191)	849 (642 to 1057)^a^	.02	0.38
Accuracy (%)	83.8 (72.7 to 95)	72.8 (54.9 to 90.8)	69.1 (43.5 to 94.7)	.06	0.30

a *P*=.01 compared to the control session. *P*=.02 compared to the traditional games session.

## Discussion

### Principal Findings

This study was designed to analyze the effects of a single session of exergames and active traditional games on the inhibitory control of school-aged children with ASD. The results highlight that the exergame session with Just Dance 2022 led to significant improvements in children’s RTs, particularly in the incongruent condition, compared to both the sedentary control session and active traditional games. Notably, the exergame session proved more effective than the active traditional games session in enhancing inhibitory control, even with a lower exercise intensity. Importantly, the active traditional games session did not differ from the sedentary control session in terms of its effects on inhibitory control. These findings suggest that exergames could be a valuable resource in educational and clinical settings, with considerable potential for improving inhibitory control in children with ASD.

Exergaming has been recognized as an alternative practice that offers substantial benefits for children with ASD, including improvements in physical fitness, executive function, and self-perception, as well as promoting greater participation in moderate to vigorous physical activities [43]. Evidence suggests that even a single 20-minute session of exergames, such as those using platforms such as Dance Dance Revolution and Cybercycling, can lead to significant improvements in executive function in young individuals with ASD [44]. Our study supports these findings, demonstrating improvements in inhibitory control following an exergame session. These results underscore the potential of exergames as a viable alternative to active traditional games, particularly in school settings. Incorporating exergames into regular physical activity programs in schools can create a stimulating and engaging environment tailored to the specific needs of children with ASD, fostering their active participation and sustained engagement.

Previous studies suggest that moderate-intensity exercise can enhance inhibitory control in children, with exercise intensity playing a crucial role in this improvement. However, our study found that exergame sessions, despite being performed at a lower intensity (64% of HRmax), resulted in superior inhibitory control compared to traditional physical education sessions (76% of HRmax). This finding indicates that the benefits of exergames may go beyond physical intensity, potentially due to the cognitive demands involved. Exergames require children to engage in both physical and virtual environments, process multiple stimuli, and make quick decisions, thereby providing intense cognitive training. This type of training may activate brain regions associated with executive control more effectively than activities that primarily emphasize physical effort [45]. Consequently, future research should further explore the role of cognitive load during physical activities and its impact on inhibitory control and academic performance.

Integrating playful activities, such as games, into exercise sessions has been identified as a promising strategy for enhancing inhibitory control and, consequently, academic performance in neurotypical children [17]. While our study did not find statistically significant effects of active traditional games on inhibitory control in children with ASD, this approach should not be dismissed as it may still offer meaningful benefits. These games create opportunities for children with ASD to engage in social interactions, improve motor skills, and experience cooperative and symbolic play—areas where they often encounter difficulties due to challenges in communication, socialization, and behavioral flexibility [20]. These activities can help reduce social isolation and foster a sense of inclusion, providing an environment conducive to skill development alongside peers [21,22]. This underscores the importance of designing physical education classes that accommodate the specific needs of children with ASD, incorporating diverse practices that support their overall development.

This study has limitations that warrant consideration in interpreting the findings and guiding future research. The small sample size, consisting of 9 Brazilian children with ASD, limits the generalizability of the results to broader populations. Nearly half of the participants had comorbid attention-deficit/hyperactivity disorder, which may have influenced the findings due to the interaction of traits such as impulsivity with inhibitory control measures. Additionally, the lack of detailed information regarding the severity of ASD symptoms and participants’ medication use constrains the interpretation of results. The testing protocol, despite including a familiarization session to mitigate novelty effects, could not fully address practice-related biases or the risk of inattentiveness during repeated neurocognitive assessments. Furthermore, the potential for day-to-day variations in performance, particularly among children with ASD, remains a concern, even with randomization and the crossover design. The 48-hour washout period, selected based on existing literature, may not have completely eliminated carryover effects, especially given the variability in cognitive responses among this population. Another limitation relates to the personalization of the intervention. Although the choice of Just Dance 2022 was justified by its adaptability to participants’ movements and immediate feedback, fostering engagement, the lack of individual customization in song selection and difficulty progression may have impacted outcomes. Finally, while a significant improvement in RT was observed following the exergame session, this was accompanied by a nonsignificant reduction in accuracy compared to the control session, suggesting a potential trade-off between speed and accuracy.

The findings of this study hold significant implications for educational and clinical settings, as they demonstrate that exergaming-based exercise activities positively impact inhibitory control in children with ASD. This is particularly important because it highlights the potential of incorporating these activities into school environments, offering opportunities for both cognitive and physical development in an inclusive and adaptive setting. Integrating exergames into the school curriculum can support the academic and social growth of these children while encouraging more active and meaningful participation in physical activities, which are vital for their overall well-being. Future research should address these limitations by incorporating larger and more diverse samples, stratifying participants based on comorbidities, collecting detailed clinical data, exploring longer washout periods, and implementing individualized interventions to ensure more robust and reliable findings. Additionally, further studies should investigate possible trade-offs between speed and accuracy in inhibitory control tasks to enhance our understanding of the observed effects.

### Conclusions

In conclusion, even a single session of the Just Dance 2022 exergame showed a significant and promising benefit for improving inhibitory control in school-aged children with ASD. The inclusion of exergames in the school routine holds great potential, as physical activity has been shown to enhance executive functions. Despite this, the use of exergames remains uncommon in many physical education programs. These games, however, can serve as an innovative pedagogical tool to enhance executive functions and help children with ASD meet recommended levels of healthy physical activity. Moreover, the use of exergames does not necessarily require a video game console; they can be implemented by projecting internet-available videos in the classroom, making them more accessible and feasible for widespread use.

## Supplementary material

10.2196/65562 Multimedia Appendix 1 Protocols of the experimental sessions.

10.2196/65562 Checklist 1 CONSORT (Consolidated Standards of Reporting Trials) checklist.

## Abbreviations

ASD: autism spectrum disorder
CONSORT: Consolidated Standards of Reporting Trials
*DSM-5*: *Diagnostic and Statistical Manual of Mental Disorders, Fifth Edition*
HRmax: maximum heart rate
KTK: Kӧrperkoordinationstest für Kinder
ReBEC: Brazilian Registry of Clinical Trials
RT: reaction time
