# Supplementary material for: Effects of a Session of Exergames and Traditional Games on Inhibitory Control in Children With Autism Spectrum Disorder: Randomized Controlled Crossover Trial
Source: JMIR Serious Games. 2025 Mar 5;13:e65562. doi: 10.2196/65562 (PMC11900902; doi:10.2196/65562)
Supplement: Multimedia Appendix 1 [file games-v13-e65562-s001.docx]

**Table S1.** Songs used in the exergame session with Just Dance 2022.

| Song Title | Artist | Link | Duration (min:sec) | Level |
| --- | --- | --- | --- | --- |
| Mood | 24kGoldn Ft. Iann dior | <https://youtu.be/HG_zpcyyWvI> | 2:23 | Light |
| Levitating | Dua Lipa | <https://youtu.be/CDVnoU1Q_Zg> | 3:34 | Medium |
| Freed from desire | GALA | <https://youtu.be/ohRMRK3DTdY> | 3:30 | Medium |
| Believer | Imagine Dragons | <https://youtu.be/aoIh9zw5_Ms> | 3:28 | Difficult |
| Last friday night | Katty Perry | <https://youtu.be/ctzZDDrHNeQ> | 4:33 | Difficult |
| Total time of songs | | | 20:50 |  |
| Total rest time between songs | | | 1:15 |  |
| Total intervention time | | | 22:05 |  |

**Table S2.** Activities used during the session with active traditional games.

| Games | Description | Duration (min:sec) | Level |
| --- | --- | --- | --- |
| Statue of the body parts | Teach the children the lyrics of the selected songs, explain that they should sing and move according to the music, when the music stops the teacher will speak two parts of the body through which they should come together (e.g. elbow on the knee, one student should put elbow on the knee of another student). | 5:00 | Light |
| Save yourself with a hug | Explain to the students that one or more students will be the catchers, the others will have to run away and, in order not to be caught, they will have to hug their classmate, releasing soon after (you can stipulate the hug time of 5 minutes), whoever is caught becomes the catcher.  Variations:  Allow hugs only between girls and boys.  I only allow hugs between girls with girls and boys with boys.  Hugs from three, four, five students. | 5:00 | Medium |
| Hula Hoop Dance | Gather the class around hula hoops, arranged in a circle, in the middle of the court, turn on a song in fast or slow rhythm (varying the rhythms) and ask them to move through the hula hoops in rhythm with the music; At each interval of the song, a hula hoop is taken out and the student who does not receive a hula hoop must enter a hula hoop belonging to another classmate. At the end there will only be two hula hoops, the students spin around one and when the song ends, they enter the other hula hoop. | 5:00 | Difficult |
| Even-Odd Tag | The class will be divided into two teams (one team will be called “even” and the other “odd”). The activity will take place on a volleyball court. The teams will stand in the middle of the court, with one team back-to-back. The teacher will say a number, if it is “even”, the students of the even team must run after the odd team, which must run away to the end of the volleyball court. When students on the odd team cross the baseline, they can no longer be caught. Each student caught will move on to the other team. When all students have passed the bottom line, they should return to the activity start line and then restart the activity with the teacher saying another number. The teacher can do the activity using mathematical operations and the result of this will be considered as the beginning of the activity. | 5:00 | Difficult |
| Total playing time | | 20:00 |  |
| Total rest time between games | | 2:05 |  |
| Total intervention time | | 22:05 |  |
